# Trastuzumab improves locoregional control in HER2-positive breast cancer patients following adjuvant radiotherapy

**DOI:** 10.1097/MD.0000000000004230

**Published:** 2016-08-12

**Authors:** Lu Cao, Gang Cai, Fei Xu, Zhao-Zhi Yang, Xiao-Li Yu, Jin-Li Ma, Qian Zhang, Jiong Wu, Xiao-Mao Guo, Jia-Yi Chen

**Affiliations:** a Department of Radiation Oncology, Fudan University Shanghai Cancer Center; b Department of Radiation Oncology, Ruijin Hospital, Shanghai Jiaotong University School of Medicine; c Department of Breast Surgery, Fudan University Shanghai Cancer Center, Shanghai, China.

**Keywords:** adjuvant radiotherapy, breast cancer, HER2+, locoregional benefit, trastuzumab

## Abstract

The benefit of adjuvant trastuzumab in disease-free and overall survival for human epidermal receptor 2–positive (HER2+) breast cancer patients is well established. However, the effect of trastuzumab on locoregional control remains unclear, particularly in patients treated with adjuvant radiotherapy (RT). In this study, we investigated the locoregional benefit of trastuzumab in patients with HER2+ breast cancer after adjuvant RT.

Using a single institutional database, we identified 278 patients with stage II/III invasive HER2+ breast tumors receiving adjuvant RT between January 2008 and July 2011. We compared the locoregional outcomes of 134 patients who received trastuzumab to 144 patients without trastuzumab within the same period. Clinical and biological factors that might impact on the locoregional benefit of trastuzumab were also assessed.

At the median follow-up of 45 months, trastuzumab significantly lowered the risk of locoregional recurrence (LRR) with a 3-year LRR rate of 2.4% versus 7.5% for the cohort with and without trastuzumab (*P* = 0.019). Trastuzumab was associated with a more significant locoregional benefit in the hormone receptor–positive (HR+)/HER2+ subgroup, with a 3-year LRR of 0% versus 6.7% in the cohort with and without trastuzumab (*P* = 0.027). For HR−/HER2+ breast tumor patients, the 3-year LRR rate was still lower for the cohort with trastuzumab (4.7% vs 8.6%). However, statistical significance was not found (*P* = 0.179). Both univariate and multivariate analyses confirmed that trastuzumab treatment was the only significant predictive factor for LRR (hazard ratio, 4.05; 95% confidence interval, 1.07–15.35; *P* = 0.039).

Adjuvant trastuzumab in addition to RT is associated with significant reduced LRR risk in HER2+ breast cancer.

## 1 Introduction

Randomized trials have proved that radiotherapy (RT) is an important therapeutic strategy in patients receiving breast-conserving surgery and high-risk node-positive patients receiving mastectomy. Adjuvant RT not only significantly reduces the risk of locoregional recurrence (LRR) but also improves the breast cancer–specific survival and overall survival. [^1^ ^2^ ^3^] As risk category was defined by traditional tumor and nodal status in these trials, facing the background of increasing modern biological information of breast cancer, whether more effective systemic therapy will influence the therapeutic outcome of adjuvant RT has become an area of interest.

Smith et al ^[4]^ identified that 15% to 25% of their breast tumor cohort overexpressed the human epidermal receptor 2 (HER2). Trastuzumab (Herceptin; Roche, Basel, Switzerland) is a humanized mouse monoclonal antibody that specifically blocks the activity of HER2, which has been shown to improve both disease-free and overall survival in node-positive and high-risk node-negative HER2+ breast cancer patients. ^[5]^ However, there are much fewer reports concerning the locoregional benefit of trastuzumab. In HER2-overexpressed breast cancer patients, adjuvant trastuzumab and RT are often indicated jointly in clinical practice. Harris et al ^[6]^ reported that HER2 overexpression was associated with the radioresistance of breast cancer cells, while trastuzumab has been found to reverse the radioresistance,[ ^7^ ^8^] which suggests a potential radiosensitizing effect in the treatment of HER2+ breast tumors.

Hugh et al ^[9]^ reported that about 61% of HER2+ breast tumors also express estrogen receptors (ERs) and/or progesterone receptors (PRs). A preclinical report by Pietras ^[10]^ identified that HER2 can alter the phosphorylation of ER and the biological activity of ER-dependent signaling networks in breast cancer. Park et al ^[11]^ found that ER status could influence the recurrence pattern of HER2+ breast tumors. To date, it is unclear whether a relationship exists between hormone receptor (HR) status and the locoregional benefit of trastuzumab in addition to adjuvant RT.

In this retrospective study, we analyzed the locoregional benefit of HER2+ breast cancer patients receiving RT with or without adjuvant trastuzumab, and assessed the impact of clinical and biological information, especially HR status, on the locoregional benefit of trastuzumab under this background.

## 2 Materials and methods

### 2.1 Patient selection

Medical records of patients with pathologically confirmed invasive HER2+ invasive primary breast cancer who received adjuvant RT for stage II/III breast cancer (TNM/AJCC Tumor Staging 7th Edition) between January 2008 and July 2011 in Fudan University Shanghai Cancer Center were reviewed. Initial tumor stage was clinically assessed in patients treated with neoadjuvant chemotherapy and pathologically for patients who underwent surgery. Patients who had simultaneous contralateral breast cancer, prior or concurrent malignancy (except nonmelanoma skin cancer), or an unknown HR status were excluded. In total, 278 patients met our inclusion criteria and were enrolled. This study was approved by Fudan University Shanghai Cancer Center Review Board and waiver of consent was obtained.

### 2.2 Assessment of HER2 and hormone receptor status

A positive HER2 status was defined by an expression level intensity of 3+ using immunohistochemistry or a gene amplification ratio >2.2 by fluorescence in situ hybridization. ER and PR statuses were assessed by immunohistochemistry. Nuclear staining ≥10% was considered a positive result. All tumor samples were examined by a pathologist.

### 2.3 Treatment with trastuzumab and adjuvant RT

Out of 278 patients, 134 (48.2%) received trastuzumab for an average of 52 weeks (range, 6–75 weeks). Of those patients, 70 received concurrent administration of trastuzumab with RT. In total, 102 patients completed 1-year trastuzumab therapy. Trastuzumab was discontinued because of cardiac toxicity in 1 patient. The remaining 31 patients discontinued trastuzumab due to financial hardship.

Only 29 of the 74 patients who received neoadjuvant chemotherapy had trastuzumab-contained regimen. Adjuvant chemotherapy was administered to 97.8% (272 of 278) patients, with 66 patients also receiving trastuzumab concurrent with chemotherapy. Of the 144 patients with HR+/HER2+ tumors, 143 (99.3%) received hormonal therapy.

All patients were treated with external beam RT (25 fractions of 50 Gy) to the ipsilateral whole breast or chest wall followed by a tumor bed boost of 10 Gy. Field borders of tangent fields and delineation of target fields were arranged according to the Radiation Therapy Oncology Group criteria. ^[12]^ At the discretion of the radiation oncologist, node-positive patients received radiation (25 fractions of 50 Gy) to the regional lymph nodes, which included the supraclavicular nodes only in 138 patients, plus internal mammary nodes in 90 patients. Fifteen patients with positive axillary lymph nodes did not receive regional node irradiation (RNI).

### 2.4 Patient follow-up

Imaging and clinical examination were done every 3 months for the first 2 years after RT, and then, every 4 to 6 months during the third, fourth, and fifth years and annually thereafter. Patients who received follow-up assessments outside of our institution were contacted by phone for information.

LRR was defined as any recurrence within the ipsilateral breast, chest wall, and ipsilateral axillary, internal mammary, infraclavicular, or supraclavicular lymph nodes, regardless of systemic disease status. Other reported outcomes included distant recurrence-free survival (DRFS) and recurrence-free survival (RFS). Distant recurrence included all sites of recurrence except locoregional relapses. Recurrence included LRRs and/or distant recurrences. The follow-up period was calculated from the date of cancer diagnosis to the date of the first event or the last confirmed date of a breast cancer disease-free status.

### 2.5 Statistical analyses

Comparisons of clinical, pathologic, and treatment-related characteristics were performed using the Pearson χ^2^ test for categorical variables and 2-sample *t* test for continuous variables. Log-rank analysis was used for group comparisons on the Kaplan–Meier survival curve. A multivariate Cox proportional hazards regression identified predictive factors for LRR. Only the variables that showed evidence of association (*P* < 0.20) in the univariate analysis were tested in the multivariate analysis. Adjusted hazard ratios with 95% confidence intervals (CIs) were reported. All tests were 2-sided and *P* < 0.05 was considered statistically significant. All statistical analyses were performed using SPSS software version 16.0.

## 3 Results

### 3.1 Patient and clinical characteristics

The median age of the 278 patients was 48 years (range, 24–77 years). The proportion of fluorescence in situ hybridization test was significantly higher in the cohort with trastuzumab (*P* < 0.001). Patients who received trastuzumab were slightly younger in age (46 vs 50 years) and significantly less likely to accept anthracycline-based chemotherapy in neoadjuvant or adjuvant phase compared to the cohort without trastuzumab. One specific finding was that there existed a significant increase in the number of patients being treated with trastuzumab from 2008 to 2011 (*P* = 0.001) (Table 1).

**Table 1:**
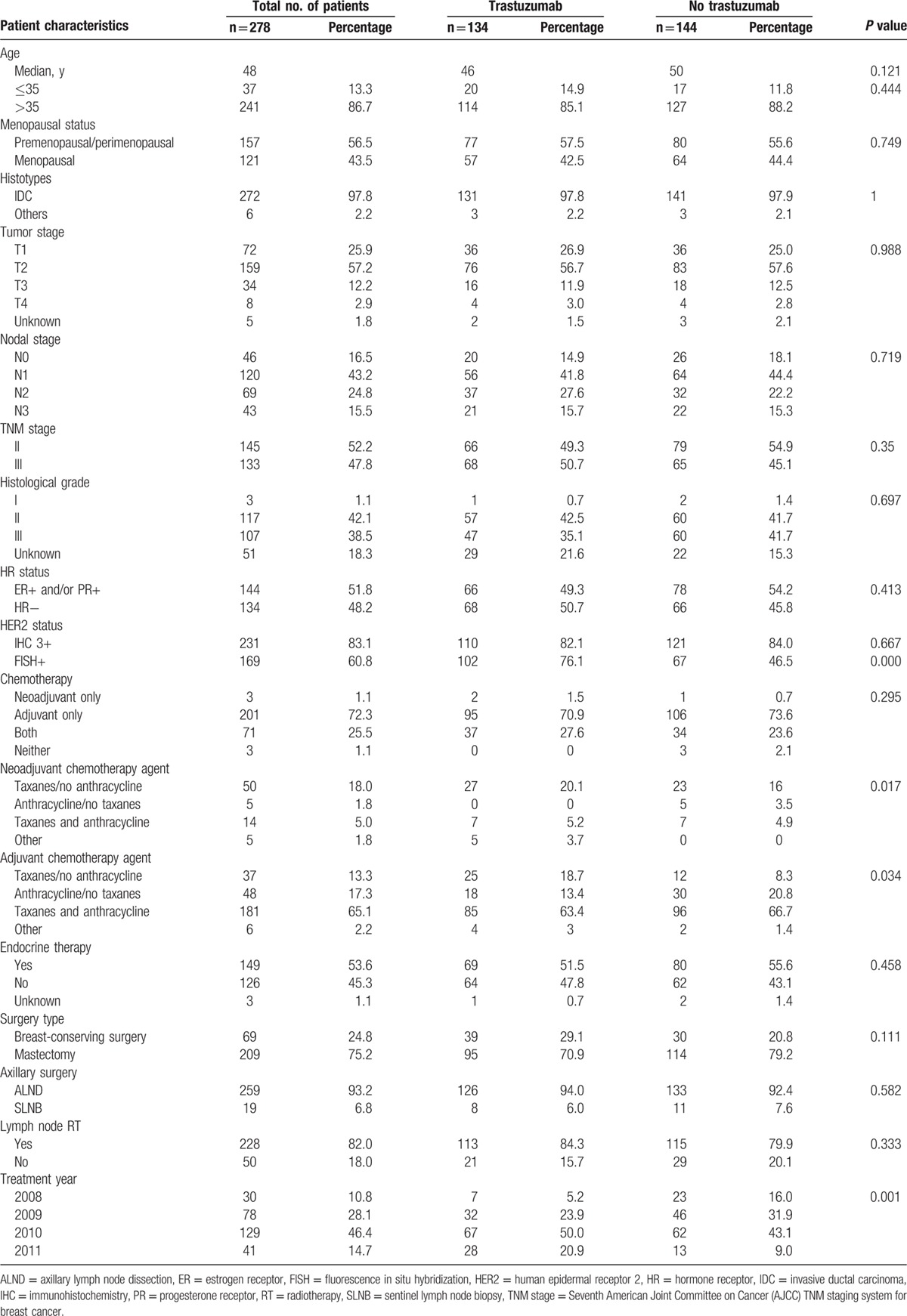
Clinical characteristics of patients with HER2-positive breast cancer.

### 3.2 Locoregional recurrence outcomes

The median follow-up period was 44 months (range, 15–70 months) for the trastuzumab cohort and 45 months (range, 12–73 months) for the cohort without trastuzumab. During the follow-up of the whole cohort, 3 of 134 in the trastuzumab cohort and 13 of 144 in the non-trastuzumab cohort were diagnosed with LRRs, which included 10 isolated LRRs, 5 with synchronous distant recurrence and 1 supraclavicular recurrence subsequent to diagnosis of bone metastasis in 5 months. The recurrence sites of these 16 patients are detailed in Table 2.

**Table 2:**
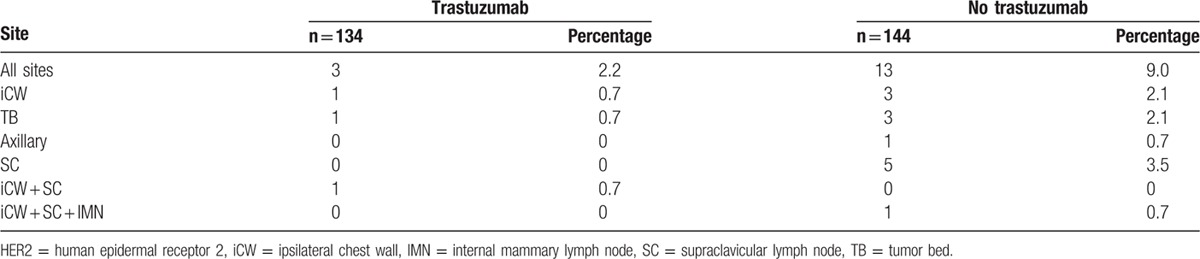
Locoregional recurrence sites of patients with HER2-positive breast cancer.

The median time to a LRR was 16 months (range, 16–36 months) in the trastuzumab group and 29 months (range, 8–54 months) in the no trastuzumab cohort (*P* = 0.552).

**Figure 1:**
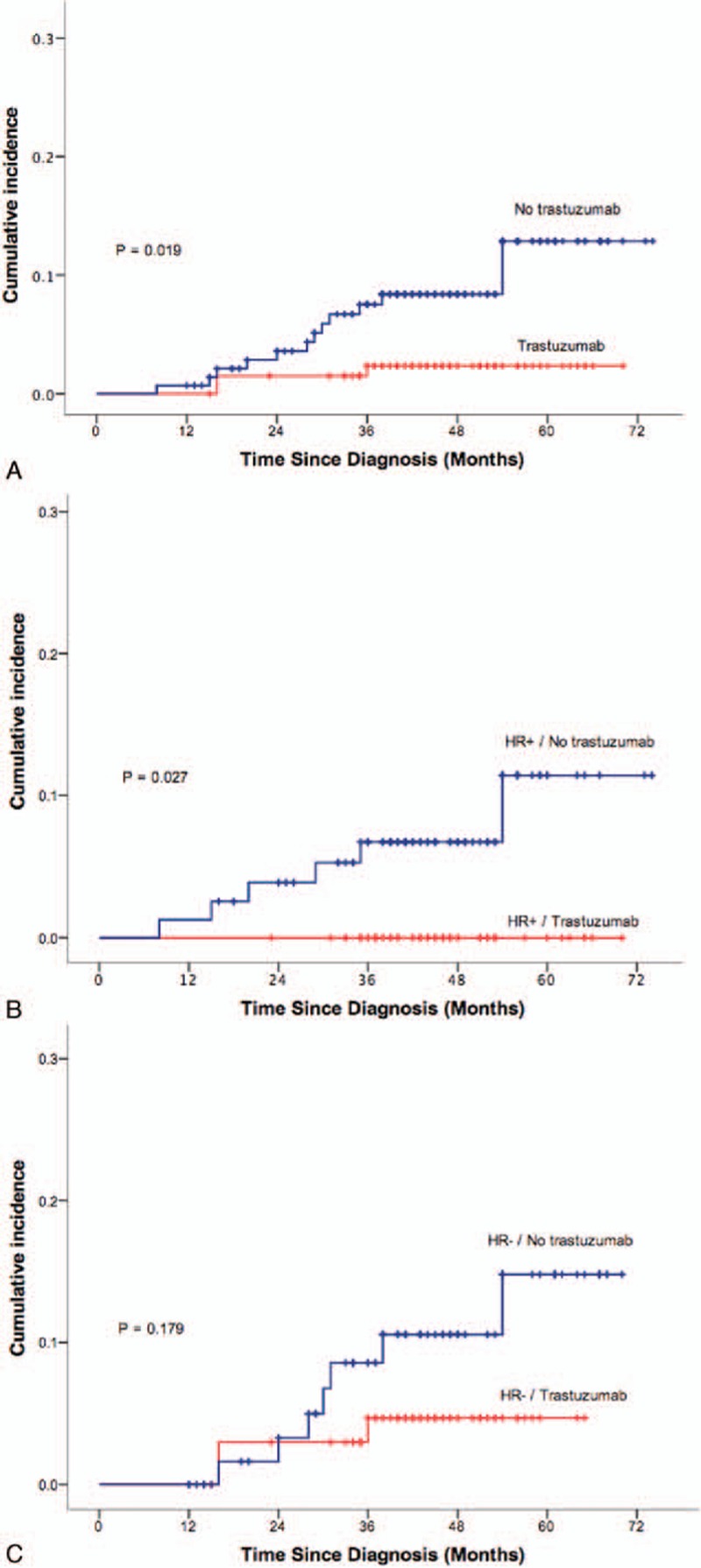
Kaplan–Meier survival curves showing LRR for all patients. (A) Survival for the total patient cohort. *X*-axis is time since diagnosis in months; *Y*-axis, probability of LRR. Red line indicates trastuzumab treatment and blue line no trastuzumab. (B) Survival for patients with HR+/HER2+ breast tumors with and without trastuzumab therapy. (C) Survival for patients with HR−/HER2+ breast tumors with and without trastuzumab therapy. *P* value is based on log-rank analysis. HER2 = human epidermal receptor 2, HR = hormone receptor, LRR = locoregional recurrence.

As shown in Fig. 1A, patients who declined trastuzumab therapy had significantly higher LRR risk. The 3-year LRR rate for this cohort was 7.5% compared to 2.4% for the cohort who had trastuzumab therapy (*P* = 0.019). Trastuzumab also reduced the isolated LRR from 4.6% in trastuzumab cohort to 2.4% in the non-trastuzumab cohort (*P* = 0.249). It was associated with reduced LRR rate (3-year LRR rate of 2.1% vs 6.6%, *P* = 0.086, and 3.1% vs 10.9%, *P* = 0.073, respectively) in patients receiving mastectomy and receiving breast-conserving surgery.

In univariate analysis (Table 3), trastuzumab was the only significant factor for LRR. RNI was also associated with a borderline significant reduction of LRR (3-year LRR rate 4.2% vs 8.1%, *P* = 0.06). Of 8 patients developing regional recurrences (RRs), 4 in the cohort of 228 patients were treated with RNI and 4 in the cohort of 50 patients were not treated with RNI. In multivariate analysis, trastuzumab treatment remains the only significant prognostic factor for LRR (hazard ratio, 4.05; 95% CI, 1.07–15.35; *P* = 0.039; Table 4).

**Table 3:**
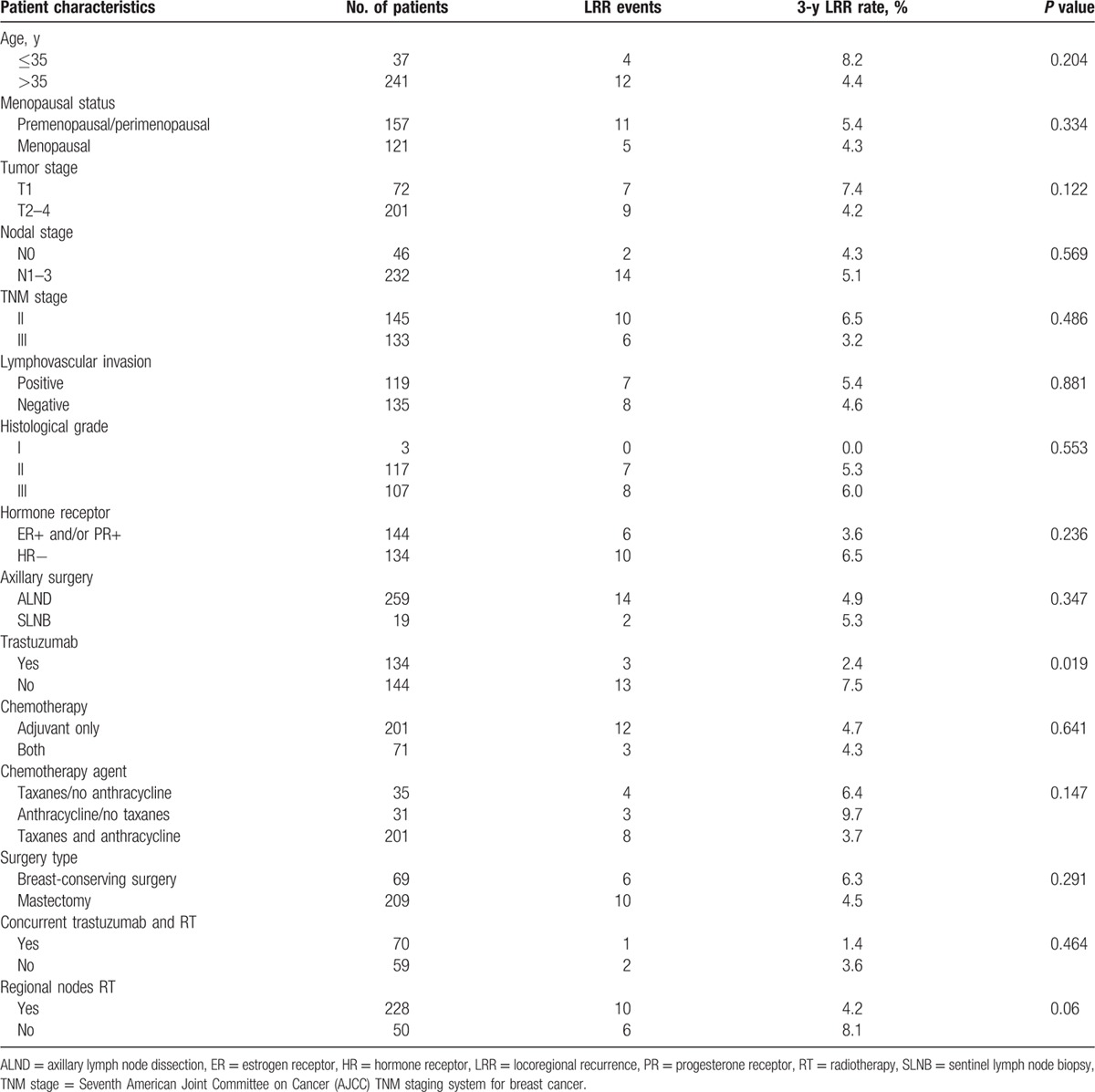
Univariate analysis of potential prognostic factors for locoregional recurrence.

**Table 4:**
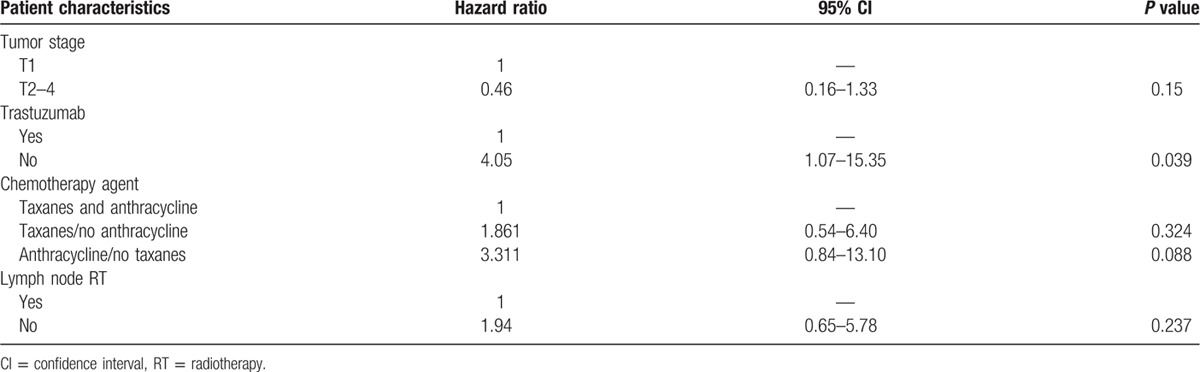
Multivariate analysis of prognostic factors for locoregional recurrence.

### 3.3 Association of hormone receptor status with LRR

Of the 144 patients with HR+/HER2+ breast tumors, 66 patients (45.8%) received trastuzumab. There were 6 LRRs in the non-trastuzumab cohort; the median time to recurrence was 25 months (range, 8–54 months). No patients in the trastuzumab cohort had LRR (Fig. 1B). The 3-year LRR rate was 0% for the trastuzumab cohort versus 6.7% for the cohort without trastuzumab therapy (*P* = 0.027).

Of the 134 patients with HR−/HER2+ breast tumors, 50.7% received trastuzumab. There were 3 LRRs in the trastuzumab group (median time to recurrence was 16 months; range, 16–36 months) and 7 recurrences in the non-trastuzumab group (median time to recurrence, 30 months; range, 16–54 months). The 3-year LRR rate was 4.7% in the trastuzumab group and 8.6% in the group without trastuzumab. However, the difference did not reach statistical significance (*P* = 0.179) (Fig. 1C).

### 3.4 DRFS and RFS outcomes

During follow-up, 49 recurrences were identified in the overall population, including 43 distant recurrence events. The 3-year DRFS and 3-year RFS were 86.4% (95% CI, 90.5–82.2%) and 84.6% (95% CI, 88.9–80.3%), respectively. Patients receiving trastuzumab had a significantly improved DRFS (3-year DRFS rate of 95.5% vs 78.0%; *P* < 0.001) and RFS (3-year RFS rate of 93.9% vs 75.9%; *P* < 0.001). The median time to recurrence was 29 months (range, 16–51 months) in the trastuzumab group and 24 months (range, 8–54 months) in the non-trastuzumab group. During follow-up, there were 6 and 1 breast cancer deaths in the non-trastuzumab cohort and trastuzumab cohort, respectively.

## 4 Discussion

To our knowledge, this is the one of first studies to assess the impact of adjuvant trastuzumab on the risk of local and regional tumor recurrences in HER2+ breast cancer patients treated with adjuvant RT. Our finding suggests that trastuzumab in addition to adjuvant RT is associated with significantly reduced LRR risk in patients with operable HER2+ breast cancer. Patients with HR+/HER2+ breast tumors were found to have a greater benefit in locoregional control from trastuzumab.

The overall survival benefit of trastuzumab has been well established. In this study, we found that trastuzumab, in addition to adjuvant RT, had a significant benefit in the locoregional control. Although much less reported, locoregional benefits of trastuzumab in combination with RT were still suggested in a few previous studies. In a retrospective study of patients with T1-T2N0, HER2+ breast cancer, Kiess et al ^[13]^ reported a marked difference in 3-year LRR rate with and without adjuvant trastuzumab (10% vs 1%) following adjuvant whole breast RT. In the 2011 HERA trial, where majority of the patients were treated with adjuvant RT, 1 year of adjuvant trastuzumab reduced the 4-year LRR rate from 5.9% (100 events) to 4.6% (79 events). ^[14]^ Following a 47-month median follow-up period, results of the FNCLCC-PACS 04 trial showed that trastuzumab was associated with an absolute reduction in LRR rates from 3.4% (9 events) to 2.7% (6 events). ^[15]^ In the FNCLCC-PACS 04 trial, adjuvant RT was administered in at least 336 patients (63.6%) undergoing breast-conserving surgery.

In preclinical studies, synergistic cell-killing effect of RT and blockade of the HER2 signaling pathway has been well established.[ ^16^ ^17^] The additional locoregional benefit of trastuzumab in addition to adjuvant RT in our study suggests that there exists at least additive effect of ionizing irradiation and HER2 blockade therapy in clinical scenario.

In our study, patients with HR+/HER2+ breast tumors seemed to have a greater locoregional benefit from trastuzumab. The locoregional benefit of trastuzumab as it relates to HR status is rarely mentioned in previous studies. To our knowledge, a retrospective study by Kim et al ^[18]^ is the only published work focusing on this issue, which shows that the locoregional benefit of trastuzumab was restricted to patients with tumors positive for ERs and/or PRs (HR+/HER2+). Slightly different from ours, only about 70% of patients were treated with adjuvant RT in this study. Although limited, aromatase inhibitors and tamoxifen have been reported to enhance radiosensitivity in several studies.[ ^19^ ^20^] Therefore, one can hypothesize the synergistic effect of hormone therapy and trastuzumab in improving locoregional control. In addition, increasing preclinical data suggest that there exists cross talk between hormonal receptor and growth factor receptors.[ ^10^ ^21^] Such cross talk can lead to synergistic tumor progression, differential sensitivity to therapies, and various patterns of tumor recurrence.[ ^11^ ^22^] Dual inhibition of hormonal receptor and growth factor receptor pathway may block such synergistic effect. A study by Peterson et al ^[23]^ showed that a HER2 monoclonal antibody (MA 7.16.4) was more effective in inhibiting cell growth in the presence of tamoxifen in a radiation-induced breast tumor model. In our study, all HR+/HER2+ tumor patients received hormone therapy, which might explain the increased locoregional control associated with trastuzumab observed in this group.

Several studies have reported that the benefit from adjuvant RT as it relates to LRR risk and breast cancer mortality ratios is more favorable in patients with low- or intermediate-risk disease compared to those with a high-risk disease.[ ^2^ ^3^ ^24^ ^25^ ^26^] A post hoc analysis of the Danish PMRT study revealed that the LRR risk versus mortality ratio was 1 death prevented for each avoidance of local tumor recurrence in patients with good prognostic markers (HER2−, HR+, early stage, and low grade), while there was no reduction in breast cancer mortality in patients with a poor prognosis. ^[25]^ It was in patients with HR+/HER2− or HR+/HER2+ tumors that the overall survival benefit of PMRT was proved in that study. ^[26]^ Based on these findings, we would like to postulate that in patients with HR+/HER2+ tumors, benefit of breast cancer–specific survival via improvement in locoregional control could be better presented when trastuzumab and hormonal therapy are given.

An additional finding of our study is that RNI was associated with a reduced LRR risk. In the MA20 trail, RNI significantly reduced the risk of LRR by 41% in patients with positive axillary lymph nodes or high-risk node-negative patients (10-year LRR free survival of 95.2% vs 92.2%; *P* = 0.009). ^[27]^ The result of EORTC 22922 also found that RNI significantly improved 10-year disease-free survival (72.1% vs 69.1%, *P* = 0.04) and 10-year DRFS (78% vs 75%, *P* = 0.02) in patients with centrally or medially located tumor or positive axillary lymph nodes. ^[28]^ Although neither MA 20 nor EORTC 22922 trial considered molecular subtypes in their enrollment, other retrospective studies have provided clues on potential impact of molecular subtypes on the risk of regional node recurrence, which can be presented as the ratio of regional node recurrence versus local recurrence (LR). Voduc et al ^[29]^ reported that 10-year LR was similar to RR in patients with HR−/HER2+ breast cancer (16% vs 14%), while 10-year LR was almost twice higher than RR in patients with HR+/HER2− breast cancer (8% vs 3%). Wo et al ^[30]^ also found that HR−/HER2+ breast cancer had a higher isolated RR than HR+/HER2− disease (5-year isolated RR rate of 5.6% vs 0.3%, *P* = 0.01). In our study, 15 out of 50 node-positive patients did not receive RNI with a median of 2 positive nodes (ranging from 1 to 19). Another 10 patients with high-risk negative axillary nodes did not receive RNI. This result implies that even though the clinical significance of RNI is less established than breast/chest wall irradiation in patients with moderate number of positive axillary nodes or high-risk node-negative disease, the newly published important trials alert us to reevaluate RNI in these subgroups, especially in those presented with unfavorable molecular subtypes such as HER2 positivity.

Some inherent limitations were considered in this retrospective study. The total isolated LRR events are much less than distant events (10 vs 43) in our study. The lack of statistical difference in isolated LRR events between trastuzumab and non-trastuzumab cohorts is very much related to the limited sample size and corresponding low number of LRR events. Trastuzumab, in addition to surgery, adjuvant RT, adjuvant or neoadjuvant chemotherapy, and hormonal therapy, reduced the 3-year isolated LRR from 4.6% to 2.4%, and 3-year LRR from 7.5% to 2.4%, which is at least proportional to the reduction of 3-year DRFS from 22.0% to 14.5%. Our result will contribute to the literature that the use of trastuzumab, apart from its established efficacy to ameliorate the disease-free survival, is associated with reduced locoregional events in patients with stage II/III HER2+ breast cancer receiving comprehensive local-regional therapy. Considering the natural progression of HR+ breast cancer, ^[31]^ it is also possible that trastuzumab delays instead of prevents LRR events, and therefore a longer follow-up period as well as a larger sample size is warranted, so as to assess the locoregional and survival benefit of trastuzumab in a longer term based on a more substantial sample size.
